# The Dental Pulp, Abscesses, and Root-Filling

**Published:** 1894-08

**Authors:** Charles Keyes

**Affiliations:** Rio de Janeiro, Brazil


### THE DENTAL PULP, ABSCESSES, AND ROOT-FILLING.

BY CHARLES KEYES, D.D.S., RIO DE JANEIRO, BRAZIL.

To destroy the dental pulp painlessly, take a disk made of No. 60 tin-foil, and cut to the size of bottom of cavity into which it is to go, and depress between end of excavator handle and a piece of soft wood, or rubber pad. Fill with campho-phénique, into which is placed sufficient aristol to make a syrupy mass; to this add two or three crystals of cocaine hydrochlorate, and in the centre of this little disk of paste put about a one-hundredth part of a grain of pure årsenic (white oxide of arsenicum). Dry the cavity (after having previously cleaned out and washed with warm water) and invert the disk and contents over the exposure and fill over with temporary stopping, being careful to avoid pressure sufficient to flatten the disk on the pulp.

Since the latter part of 1889 I have used almost exclusively this method, and I cannot record a single case which gave pain, unless inflammation existed in the pulp previously to the application.

For allaying inflammation in the pulp, extending to suppuration, with or without pain, I have found nothing more effective than *thymol*, which is applied pulverized in a tin disk inverted over exposure and cavity filled with temporary stopping, having previously cleaned the cavity and washed with warm water. If pain be very severe, a drop of chloroform placed in the cavity, after the disk with thymol is adjusted, and previous to putting in temporary stopping, will usually lessen pain; otherwise thymol generally requires from ten to fifteen minutes to produce effect. In slight cases one application is usually sufficient, but in severe ones sometimes several are necessary before arsenic may be applied.

To avoid pericementitis, abscess, etc., in the removal of putrescent pulps, exclude absolutely the entrance of saliva, use clean instruments, flood cavity with some good antiseptic, and at first sitting remove all of the pulp possible without letting any of the matter or the end of the broach pass beyond the apex. The patient should be told to give notice on first sign of sensation, as the main point is to avoid, under all circumstances, the passage of anything into the tissues beyond the end of the root. This done, follow with loose dressing of campho-phénique containing about ten per cent. of aristol. If a second dressing be necessary, use oil of cassia with aristol in same proportion as above. Vapors of the former are less likely to produce irritation than those of the latter. In using peroxide of hydrogen, pyrozone, or natrium and kalium, care must be taken to keep the entrance to canals from clogging, else the gases set free may pass the other way and carry with them matter beyond the apex, which must be avoided.

To treat abscess without fistula ("blind abscess"): After the canals have been thoroughly freed from septic matter, pump into the abscess campho-phénique, with ten per cent. aristol, until the patient can feel it, leaving very loose dressing in the root with only sufficient cotton to keep out food (pure aristol incorporated into cotton anywhere exposed in the mouth will keep it "sweet" much longer). Subsequent treatment consists in keeping the canal clean and aseptic, with loose dressings, until no more pus can be found in the canal. Much trouble is frequently caused by over-treatment.

If hydrogen peroxide be used to clean out the canal and abscess, care must be taken not to allow the apex to become clogged.

For filling root-canals: Take of oxide of zinc and aristol equal parts, and oil of cassia and vaseline enough to make a soft, putty-like paste. After the canal or canals are thoroughly dried, fill the pulp-chamber with this paste, and with a hot instrument, or nerve broach with wisp of cotton wrapped around it, work into place. After the canal is filled with this mass, a gutta-percha cone, the size to fit the case, is pressed into the canal, the surplus being removed with a hot instrument. The balance of the pulp cavity is filled with any of the cements.

I have used this paste alone in filling roots of temporary teeth, and subsequently, after roots were absorbed, extracted them and found the paste, though much harder and tougher, perfectly intact, and protruding a little, somewhat like crabs' eyes. In no instance have I found any irritation caused by this paste, which I have used constantly for over three years.

